# Supplementary material for: Direct observation of DNA target searching and cleavage by CRISPR-Cas12a
Source: Nat Commun. 2018 Jul 17;9:2777. doi: 10.1038/s41467-018-05245-x (PMC6050341; doi:10.1038/s41467-018-05245-x)
Supplement: Supplementary file 1 — Supplementary Information [file 41467_2018_5245_MOESM1_ESM.docx]

**Supplementary Information**

**Direct observation of DNA target searching and cleavage by CRISPR-Cas12a**

**Yongmoon Jeon, You Hee Choi, Yunsu Jang, Jihyeon Yu, Jiyoung Goo, Gyejun Lee, You Kyeong Jeong, Seung Hwan Lee, In-San Kim, Jin-Soo Kim, Cherlhyun Jeong, Sanghwa Lee & Sangsu Bae**

*Content:*

*Supplementary Figure 1.* Relative populations of stable binding events (S2) as a function of the number of transient binding (S1) trial before S2 formation

*Supplementary Figure 2.* Characterization of dye photobleaching time in the experiment with 0.5-s time resolution

*Supplementary Figure 3.* Single-molecule FRET and PIFE experiments using doubly labelled DNAs to directly observe the release events of cleaved DNA fragment

*Supplementary Figure 4.* Dwell-time histograms for individual steps of the DNA cleavage reaction by AsCas12a RNPs

*Supplementary Figure 5.* In vitro DNA cleavage assay for determining DNA cleavage efficiency of AsCas12a with partially cognate DNAs using an agarose gel electrophoresis

*Supplementary Figure 6.* AsCas12a cannot bind to target DNAs that lack a PAM sequence

*Supplementary Table 1.* DNA duplexes and crRNA sequences used in this study

*Supplementary Table 2.* DNA oligonucleotides for plasmid cloning used in this study

**Supplementary Figure 1.** Relative populations of stable binding events (S2) as a function of the number of transient binding (S1) trial before S2 formation


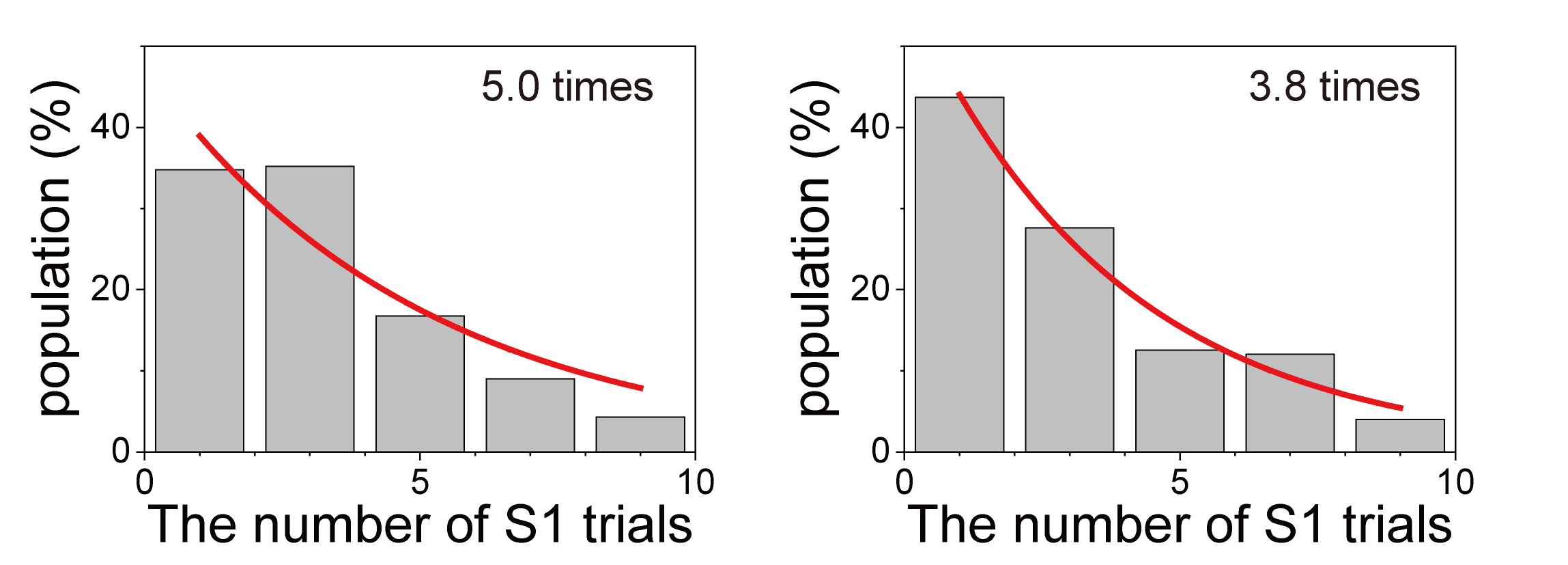


The average number of transient binding trials per a stable binding event (S2) was obtained by fitting the data to s single-exponential function. To obtain each histogram, the number of S1 trials were collected from at least more than 200 time trajectories (248 for left; 215 for right). Experiments were performed with 20 nM AsCas12a RNPs. To obtain error bar in Figure 2F, this experiment was repeated twice.

**Supplementary Figure 2.** Characterization of dye photobleaching time in the experiment with 0.5-s time resolution


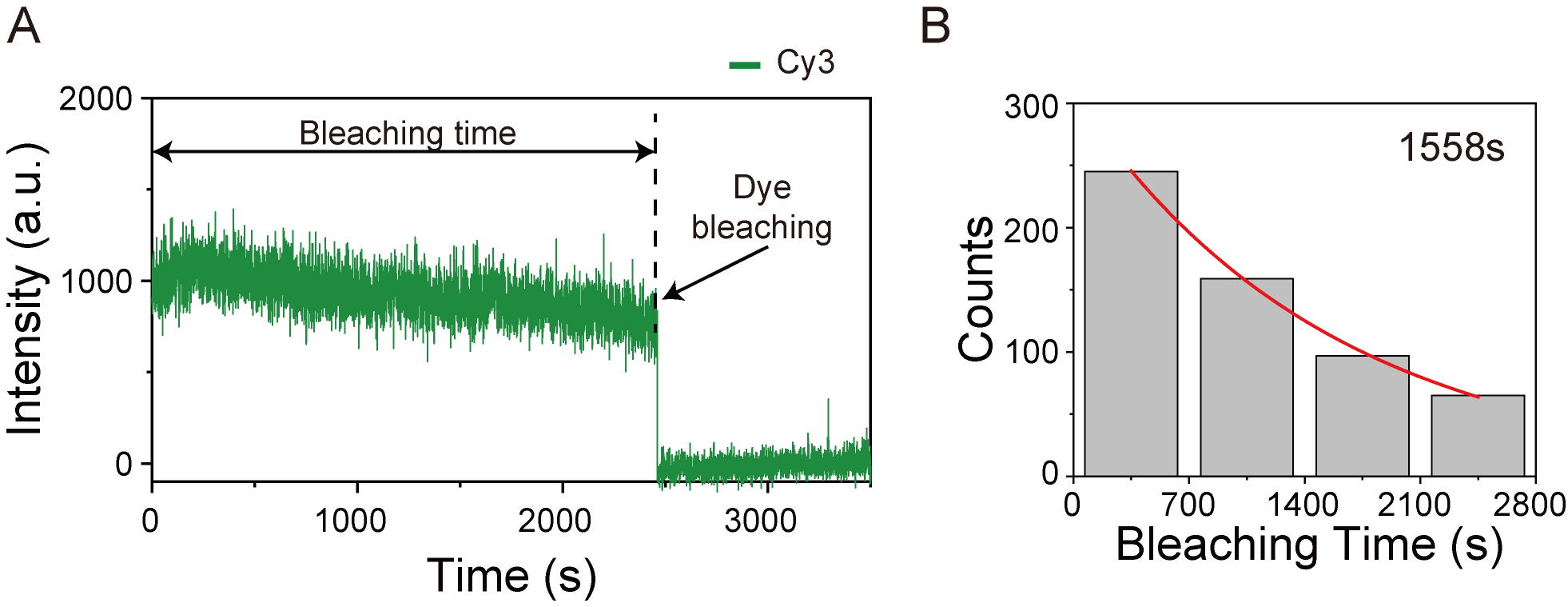


(A) Representative fluorescence intensity time trace showing a dye photobleaching event. In this measurement, the experimental condition including the excitation laser and the time resolution (0.5-s) was same as in Figure 2D. (B) Histogram of dye photobleaching time. The histogram was made from more than 500 bleaching events observed in three independent experiments with 0.5-s time resolution. The data was fit to single-exponential decay function to obtain the average dye photobleaching time (1558-s) under the experimental condition.

**Supplementary Figure 3.** Single-molecule FRET and PIFE experiments using doubly labelled DNAs to directly observe the release events of cleaved DNA fragment


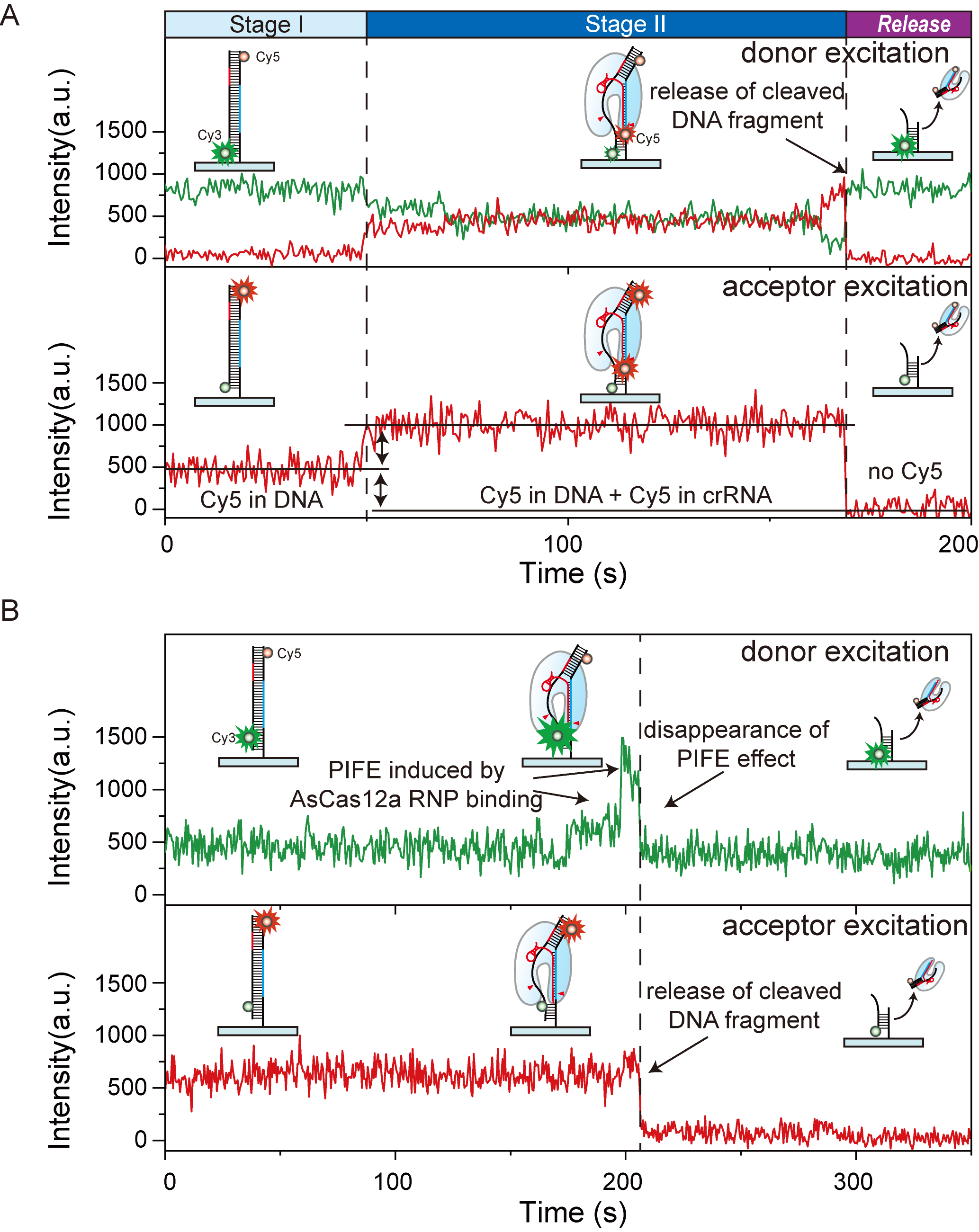


(A) Representative time traces of Cy3 fluorescence (green, top), Cy5 fluorescence (red, top) under donor excitation and Cy5 fluorescence under acceptor excitation (red, bottom) showing stage II with subsequent FRET states including DNA cleavage and release. In (A), we performed alternating-laser excitation (ALEX) FRET experiments with Cy3-Cy5 doubly labeled DNA and Cy5 labeled crRNA to simultaneouly monitor the release of both Cy5 labeled crRNA and cleaved DNA fragment. (B) Representative time traces of Cy3 fluorescence under donor excitation (green, top) and Cy5 fluorescence under accpeptor excitation (red, bottom) showing Cy3 fluorescence enhancement induced by AsCas12a RNP binding and the subsequent release event of Cy5 labeled cleaved DNA fragment. In (B), we performed the experiment termed protein-induced fluorescence enhancement (PIFE) with ALEX technique. In this experiment, the intensity of a fluorophore is increased when a protein binds in its vicinity due to the enhancement in the quantum yield of the fluorophore. In our experiment, the PIFE occurred only in Cy3, and almost not in Cy5, upon the final step before occurring the release of cleaved DNA fragment. We also found that the PIFE effect was disappeared immediately after the release of cleaved DNA fragment, which indicates that both cleaved DNA fragment and AsCas12a RNP were simultaneously released. In (A,B), experimental schemes were added above each time trace to clearly reveal our data interpretation.

**Supplementary Figure 4.** Dwell-time histograms for individual steps of the DNA cleavage reaction by AsCas12a RNPs


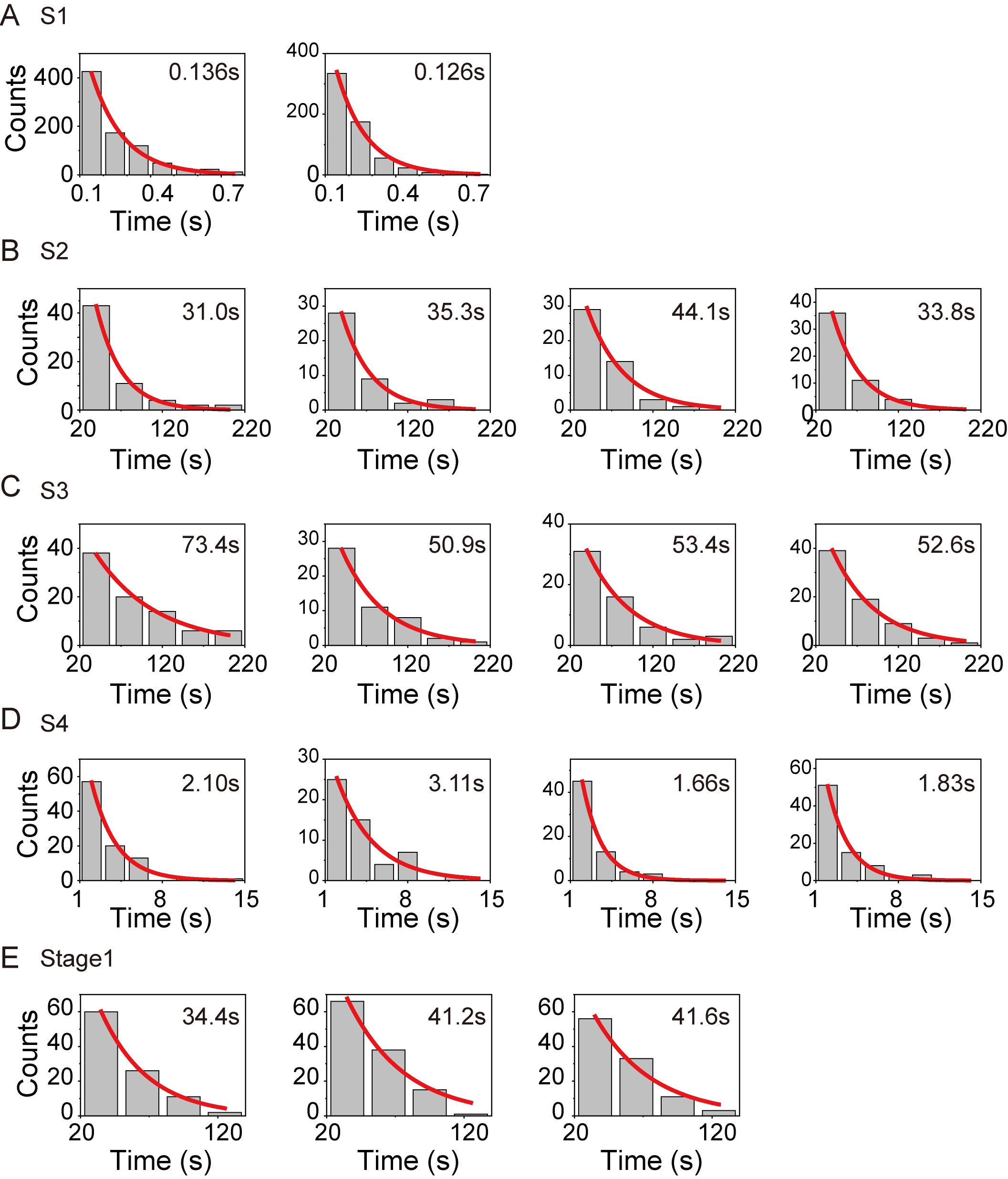


Histograms of dwell times for individual states, S1 (A), S2 (B), S3 (C), S4 (D) and stage I (E) of the cleavage reaction by WT AsCas12a RNPs. To obtain dwell time histograms for individual states, each event was collected from at least more than 64 time trajectories. Each histogram was fit to single-exponential function to obtain corresponding kinetic times. Experiments were performed with 20 nM AsCas12a RNP. To obtain standard deviations of time constants in Figure 2F, each experiment was repeated at least twice.

**Supplementary Figure 5.** In vitro DNA cleavage assay for determining DNA cleavage efficiency of AsCas12a with partially cognate DNAs using an agarose gel electrophoresis


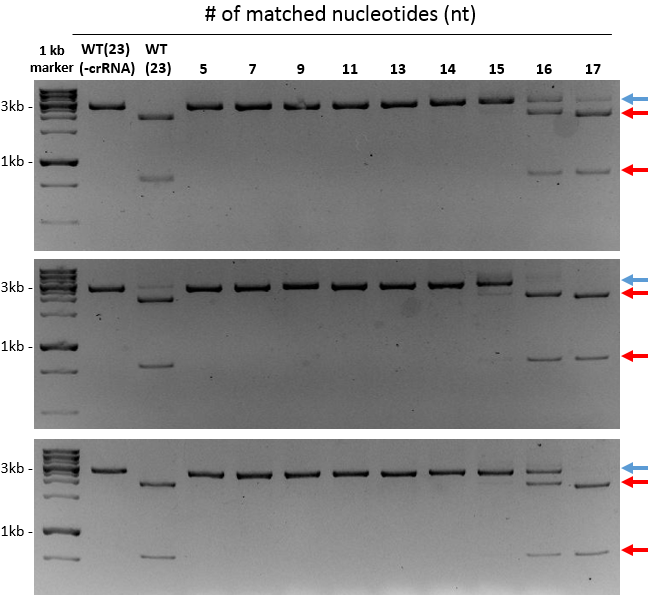


DNA cleavage efficiencies of AsCas12a with partially cognate DNAs are assessed by *in vitro* cleavage assay using agarose gel electrophoresis (Online Methods). Number of matched nucleotides with crRNA are extending from the PAM-proximal side. Substrate and cleaved products are indicated with blue and red arrows, respectively. The experiment was repeated three times. Statistics are shown in Figure 4C.

**Supplementary Figure 6**. AsCas12a cannot bind to target DNAs that lack a PAM sequence


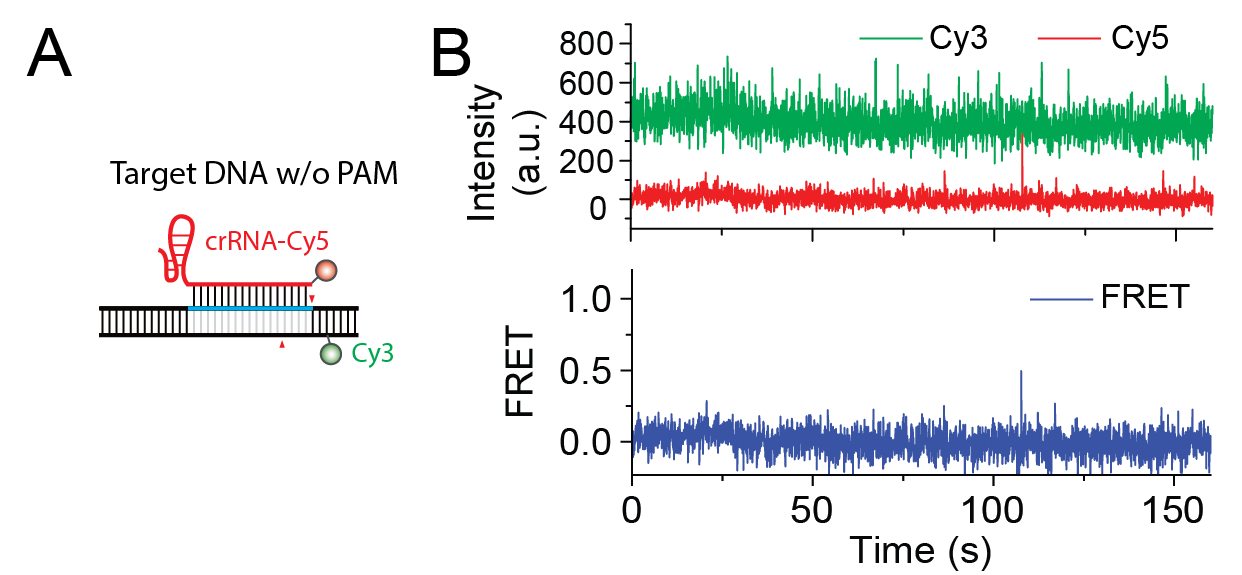


(A) Scheme of DNA duplex that containing proto spacer but lack PAM sequence. (B) Representative time trace of Cy3 (green, top), Cy5 (red, top) under donor excitation and FRET (blue, bottom) showing that AsCas12a cannot stably binding to the target sequence without PAM sequence. We analyzed 200 time traces to observe the interaction between AsCas12a and target DNA without PAM. [AsCas12a] = 5 nM, [crRNA-Cy5] = 10 nM, time resolution is 60 ms.

**Supplementary Table 1.** DNA duplexes and crRNA sequences used in this study

A. DNA sequences and modifications

| Used in | Description | Strand Sequence (5’ to 3’) |
| --- | --- | --- |
| Figure 1 | 21kb DNA | 5’- =============== 21kb DNA ===AATTCTGCACCGTGAAGTCCTCCG -3’  3’-Bio-TTCCCGCCGCTGGA=== Fragment =======GACGTGGCACTTCAGGAGGCTT-Bio-5’ |
| Figure 1G | For λ-DNA  (annealed to cosL side of λ−DNA) | 5’-AGGTCGCCGCCCAGTTACAGATTTATGGTGACGATACAAACTATAGAGTGATTTTTTTTTTTTTTTTTTTTTTTTTT  TTTTTTTTTTTTTTTTT-Bio-3’ |
| Figure 2, 3 | Target DNA (WT) | 5’-Bio-TTTTTTCACTTGACAGGCGAGTAACAGACATGGACCATCAGGAAACATTAACGTACT-3’  3’- G**T**GAACTGTCCGCTCATTGTCTGTACCTGGTAGTCCTTTGTAATTGCATGA-5’ |
| Figure 3 | NTS-Nick | 5’-Bio-TTTTTTCACTTGACAGGCGAGTAA-CAGACATGGACCATCAGGAAACATTAACGTACT-3’  3’- G**T**GAACTGTCCGCTCATT**/**GTCTGTACCTGGTAGTCCTTTGTAATTGCATGA-5’ |
|  | TS-Nick | 5’-Bio-TTTTTTCACTTGACAGGCG**/**AGTAACAGACATGGACCATCAGGAAACATTAACGTACT-3’  3’- G**T**GAACTGTCCGC-TCATTGTCTGTACCTGGTAGTCCTTTGTAATTGCATGA-5’ |
| Figure 4 | 0 match | 5’-Bio-TTCACTTGACAGGCACTCGTCTCAACGCACACTGATAGAAACATACACGTACT-3’  3’- GTGAACTGTCCGTGAGCAGAGTTGCGTGTGACTATCTTTGTA**T**GTGCATGA-5’ |
|  | 5 match | 5’-Bio-TTCACTTGACAGGCACTCGTCTCAACGCACATATCAGGAAACATTAACGTACT-3’  3’- GTGAACTG**T**CCGTGAGCAGAGTTGCGTGTATAGTCCTTTGTAATTGCATGA-5’ |
|  | 7 match | 5’-Bio-TTCACTTGACAGGCACTCGTCTCAACGCACCCATCAGGAAACATTAACGTACT-3’  3’- GTGAACTG**T**CCGTGAGCAGAGTTGCGTGGGTAGTCCTTTGTAATTGCATGA-5’ |
|  | 9 match | 5’-Bio-TTCACTTGACAGGCACTCGTCTCAACGCGACCATCAGGAAACATTAACGTACT-3’  3’- GTGAACTG**T**CCGTGAGCAGAGTTGCGCTGGTAGTCCTTTGTAATTGCATGA-5’ |
|  | 11 match | 5’-Bio-TTCACTTGACAGGCACTCGTCTCAACTGGACCATCAGGAAACATTAACGTACT-3’  3’- GTGAACTG**T**CCGTGAGCAGAGTTGACCTGGTAGTCCTTTGTAATTGCATGA-5 |
|  | 13 match | 5’-Bio-TTCACTTGACAGGCACTCGTCTCTCATGGACCATCAGGAAACATTAACGTACT-3’  3’- GTGAACTG**T**CCGTGAGCAGAGAGTACCTGGTAGTCCTTTGTAATTGCATGA-5’ |
|  | 15 match | 5’-Bio-TTCACTTGACAGGCACTCGTCTGACATGGACCATCAGGAAACATTAACGTACT-3’  3’- GTGAACTG**T**CCGTGAGCAGACTGTACCTGGTAGTCCTTTGTAATTGCATGA-5’ |
|  | 17 match | 5’-Bio-TTCACTTGACAGGCACTCGTCAGACATGGACCATCAGGAAACATTAACGTACT-3’  3’- GTGAACTG**T**CCGTGAGCAGTCTGTACCTGGTAGTCCTTTGTAATTGCATGA-5’ |
|  | 23 match | 5’-Bio-TTCACTTGACAGGCGAGTAACAGACATGGACCATCAGGAAACATTAACGTACT-3**’**  3’- GTGAACTG**T**CCGCTCATTGTCTGTACCTGGTAGTCCTTTGTAATTGCATGA-5’ |
| Figure 5C, D  (AsCas12a) | Target DNA (WT) | 5’-GGATCTCTTAAGTCGTTGAGTAACAGACATGGACCATCAGGAAACACAGTGCAGTATTAGCACTTTTT-Bio-3’  3’-CCTAGAGAA**T**TCAGCAACTCATTGTCTGTACCTGGTAGTCCTTTGTGTCACGTCATAATCGTG -5’ |
|  | Bubble | 5’-GGATCTCTTAAGTCGTTGAGTAACAGACATGGACCATCAGGAAACACAGTGCAGTATTAGCACTTTTT-Bio-3’  3’-CCTAGAGAA**T**TCAGCAACTC CTTTGTGTCACGTCATAATCGTG -5’  TATTGAGTTAAACATTTTTT |
|  | Bubble w/o PAM | 5’-GGATCTCTTAAGTCGTTGAGTAACAGACATGGACCATCAGCGGACACAGTGCAGTATTAGCACTTTTT-Bio-3’  3’-CCTAGAGAA**T**TCAGCAACTC GCCTGTGTCACGTCATAATCGTG -5’  TATTGAGTTAAACATTTTTT |
| Figure 5C (Cas9) | Target DNA (WT) | 5’- CGTGTGCTAATACTGCACTGTGACCGATGTATAATAATTGATGTCAACGAACGATGCT-**Cy3**-3’  3’-bio-TTTTTGCACACGATTATGACGTGACACTGGCTACATATTATTAACTACAGTTGCTTGCTACGATCT -5’ |
|  | Bubble | 5’- CGTGTGCTAATACTGCACTGTGACCGATGTATAATAATTGATGTCAACGAACGATGCT-**Cy3**-3’  3’-bio-TTTTTGCACACGATTATGACGTGACACTGGC TGCTTGCTACGA -5’  CCTGTTCTGCTATTCGAGGC |
|  | Bubble w/o PAM | 5’- CGTGTGCTAATACTGCACTGTGAAATATGTATAATAATTGATGTCAACGAACGATGCT-**Cy3**-3’  3’-bio-TTTTTGCACACGATTATGACGTGACACTTTA TGCTTGCTACGA -5’  CCTGTTCTGCTATTCGAGGC |
| Supplementary  Figure 2A | Doubly labelled Target DNA | 5’-Bio-TTTTTTCACTTGACAGGCGAGTAACAGACATGGACCATCAGGAAACATTAACG**T**ACT-3’  3’- G**T**GAACTGTCCGCTCATTGTCTGTACCTGGTAGTCCTTTGTAATTGCATGA-5’ |
| Supplementary  Figure 2B | Target DNA for PIFE | 5’-Bio-TTTTTTCACTTGACAGGCGAGTAACAGACATGGACCATCAGGAAACATTAACG**T**ACT-3’  3’- GTGAACTG**T**CCGCTCATTGTCTGTACCTGGTAGTCCTTTGTAATTGCATGA-5’ |
| Supplementary  Figure 5 | Target DNA  w/o PAM | 5’-Bio-TTCACTTGACAGGCGAGTAACAGACATGGACCATCAGGAAGCATAAGCGTACT-3’  3’- GTGAACTG**T**CCGCTCATTGTCTGTACCTGGTAGTCCTTCGTATTCGCATGA-5’ |

- Bio: biotin, **T** or **T**: amino modifier C6 dT labelled with Cy3-NHS mono ester (for green) or Cy5-NHS mono ester (for red) (GE Healthcare). **Cy3**: Cy3 label via amine modification.
- PAM (5’-TTTV-3’ or 5’-NGG-3’) are highlighted in yellow and DNA target sequence (complementary to crRNA guide sequence) are shown in red. Gray highlighted slash mark (/) shows nick.

B. crRNA sequences modifications

| Name | Strand Sequence (5’ to 3’) and usage |
| --- | --- |
| 80% crRNA-Cy5 | 5’-AAUUUCUACUCUUGUAGAUACGGUCGAGCGUCAGCGUCCGGG-Cy5-3’ |
|  | Used in Figure 1(B,C,D,E) |
| DNMT1-crRNA-Cy5 | 5’-AAUUUCUACUCUUGUAGAUCUGAUGGUCCAUGUCUGUUACUC-Cy5-3’ |
|  | Used in Figure 1 (F,G), Fig,2, Figure 3, Figure 4, Figure 5 |
| DNMT1-crRNA | 5’-AAUUUCUACUCUUGUAGAUCUGAUGGUCCAUGUCUGUUACUC-3’ |
|  | Used in Supplementary Figure 2A |
| Cas9-crRNA | 5’-Cy5-UGACAUCAAUUAUUAUACAUGUUUUAGAGCUAUGCUGUUUUG-3’ (used in Figure 5C) |
| Cas9-tracrRNA | 5’-GGAACCAUUCAAAACAGCAUAGCAAGUUAAAAUAAGGCUAGUCCGUUAUCAACUUGAAAAAGUGGCACCGAGUUUUUUUCGUGGCU-3’ (used in Figure 5C) |

- crRNA guide sequence (complementary to proto spacer) are shown in red.

**Supplementary Table 2.** DNA oligonucleotides for plasmid cloning used in this study

| **primer** | **sequence (5'-3')** |
| --- | --- |
| WT_TS | CACCGGAGTAACAGACATGGACCATCAGGAAA |
| WT_NTS | AAACTTTCCTGATGGTCCATGTCTGTTACTCC |
| mis_16-23_TS | CACCGCTCATTGTGACATGGACCATCAGGAAA |
| mis_16-23_NTS | AAACTTTCCTGATGGTCCATGTCACAATGAGC |
| mis_1-5_TS | CACCGGAGTAACAGACATGGACCTAGTCGAAA |
| mis_1-5_NTS | AAACTTTCGACTAGGTCCATGTCTGTTACTCC |
| mis_1-2_TS | CACCGGAGTAACAGACATGGACCATCTCGAAA |
| mis_1-2_NTS | AAACTTTCGAGATGGTCCATGTCTGTTACTCC |
| mis_5-6_TS | CACCGGAGTAACAGACATGGACGTTCAGGAAA |
| mis_5-6_NTS | AAACTTTCCTGAACGTCCATGTCTGTTACTCC |
| mis_9-10_TS | CACCGGAGTAACAGACATCCACCATCAGGAAA |
| mis_9-10_NTS | AAACTTTCCTGATGGTGGATGTCTGTTACTCC |
| mis_13-14_TS | CACCGGAGTAACAGTGATGGACCATCAGGAAA |
| mis_13-14_NTS | AAACTTTCCTGATGGTCCATCACTGTTACTCC |
| mis_17-18_TS | CACCGGAGTATGAGACATGGACCATCAGGAAA |
| mis_17-18_NTS | AAACTTTCCTGATGGTCCATGTCTCATACTCC |
| mis_18-19_TS | CACCGGAGTTTCAGACATGGACCATCAGGAAA |
| mis_18-19_NTS | AAACTTTCCTGATGGTCCATGTCTGAAACTCC |
| mis 22-23_TS | CACCGCTGTAACAGACATGGACCATCAGGAAA |
| mis 22-23_NTS | AAACTTTCCTGATGGTCCATGTCTGTTACAGC |
| WT_DNMT1_TS | CACCCACTTGACAGGCGAGTAACAGACATGGACCATCAGGAAACATTAACGTACT |
| WT_DNMT1_NTS | AAACAGTACGTTAATGTTTCCTGATGGTCCATGTCTGTTACTCGCCTGTCAAGTG |
| 5seed_TS | CACCCACTTGACAGGCACTCGTCTCAACGCACATATCAGGAAACATTAACGTACT |
| 5seed_NTS | AAACAGTACGTTAATGTTTCCTGATATGTGCGTTGAGACGAGTGCCTGTCAAGTG |
| 7seed_TS | CACCCACTTGACAGGCACTCGTCTCAACGCACCCATCAGGAAACATTAACGTACT |
| 7seed_NTS | AAACAGTACGTTAATGTTTCCTGATGGGTGCGTTGAGACGAGTGCCTGTCAAGTG |
| 9seed_TS | CACCCACTTGACAGGCACTCGTCTCAACGCGACCATCAGGAAACATTAACGTACT |
| 9seed_NTS | AAACAGTACGTTAATGTTTCCTGATGGTCGCGTTGAGACGAGTGCCTGTCAAGTG |
| 11seed_TS | CACCCACTTGACAGGCACTCGTCTCAACTGGACCATCAGGAAACATTAACGTACT |
| 11seed_NTS | AAACAGTACGTTAATGTTTCCTGATGGTCCAGTTGAGACGAGTGCCTGTCAAGTG |
| 13seed_TS | CACCCACTTGACAGGCACTCGTCTCTCATGGACCATCAGGAAACATTAACGTACT |
| 13seed_NTS | AAACAGTACGTTAATGTTTCCTGATGGTCCATGAGAGACGAGTGCCTGTCAAGTG |
| 14seed_TS | CACCCACTTGACAGGCACTCGTGTCACATGGACCATCAGGAAACATTAACGTACT |
| 14seed_NTS | AAACAGTACGTTAATGTTTCCTGATGGTCCATGTGACACGAGTGCCTGTCAAGTG |
| 15seed_TS | CACCCACTTGACAGGCACTCGTGTGACATGGACCATCAGGAAACATTAACGTACT |
| 15seed_NTS | AAACAGTACGTTAATGTTTCCTGATGGTCCATGTCACACGAGTGCCTGTCAAGTG |
| 17seed_TS | CACCCACTTGACAGGCACTCGTCAGACATGGACCATCAGGAAACATTAACGTACT |
| 17seed_NTS | AAACAGTACGTTAATGTTTCCTGATGGTCCATGTCTGACGAGTGCCTGTCAAGTG |
