# Supplementary material for: Direct observation of DNA target searching and cleavage by CRISPR-Cas12a
Source: Nat Commun. 2018 Jul 17;9:2777. doi: 10.1038/s41467-018-05245-x (PMC6050341; doi:10.1038/s41467-018-05245-x)
Supplement: Supplementary file 3 — Description of Additional Supplementary Information [file 41467_2018_5245_MOESM3_ESM.docx]

**Description of Additional Supplementary Files**

File Name: Supplementary Movie 1

Description:

Video clip showing the movement of Cy5 labelled AsCas12a RNPs along the DNA, related to Figure 1C. Upper and bottom channel show the dsDNA (Sytox orange labelled) and AsCas12a RNPs (Cy5 labelled), respectively. This clip demonstrates the entire DNA cleavage process by AsCas12a RNP from target searching via 1D diffusion to DNA release after DNA cleavage in real-time.
